# A computer-enhanced pH study of the formaldehyde–sulphite clock reaction

**DOI:** 10.1155/S146392469200018X

**Published:** 1992

**Authors:** F. T. Chau, K. W. Mok

**Affiliations:** Department of Applied Biology and Chemical Technology Hong Kong Polytechnic Hung Hom Kowloon Hong Kong

## Abstract

The formaldehyde-sulphite clock reaction was studied using an Orion SA 720 pH/ISE meter interfaced to an IBM PC. The laboratory software ‘ASYST’ was employed to facilitate data acquisition and data treatment. Experimental pH profiles thus obtained for the first time were simulated by invoking a theoretical model based on the reaction mechanism suggested by Burnett [1]. The variation of rate constants with compositions of reaction mixtures was also discuseed in light of the empirical expression proposed by Bell and Evans [2] for instantaneous rate constant of the clock reaction.


Journal of Automatic Chemistry, Vol. 14, No. 3 (May–June 1992), pp. 79–83

### A computer-enhanced pH study of the formaldehyde-sulphite clock reaction

**F. T. Chau and K. W. Mok**
*Department of Applied Biology and Chemical Technology, Hong Kong Polytechnic, Hung Hom, Kowloon, Hong Kong*

*The formaldehyde-sulphite clock reaction was studied using an Orion SA 720 pH/ISE meter interfaced to an IBM PC. The laboratory software 'ASYST' was employed to facilitate data acquisition and data treatment. Experimental pH profiles thus obtained for the first time were simulated by invoking a theoretical model based on the reaction mechanism suggested by Burnett [1]. The variation of rate constants with compositions of reaction mixtures was also discuseed in light of the empirical expression proposed by Bell and Evans [2] for instantaneous rate constant of the clock reaction.*

### Introduction

The formaldehyde-sulphite reaction has been investigated for decades [1–8]. It has been found that when the two reagents mix with each other, there is a small increase in pH in the reacting system. However, a dramatic rise in pH occurs when the reaction approaches completion. In previous studies on this clock reaction, the time intervals required for the mixing reagents to reach end-points were measured manually using colour indicators. Wagner [3] proposed a mechanism for the process, but it was revised later by Burnett [1]. Based on the modified reaction path, Warneck [9] suggested a theoretical model to explain pH profiles of the formaldehyde-sulphite system; no comparison was made, however, between calculated and experimental data. Moreover, the treatment is good for resulting solutions with pH values less than eight. In the present work, a real-time pH measuring system, with a potentiometer connected to an IBM PC [10], was employed to monitor variations of the pH quantity within the formaldehyde-sulphite reaction. In this way errors introduced through the use of colour indicators can be eliminated. The entire pH profiles of the process, as well as end-points, were obtained. A modified formulation based on that proposed before (Warneck [9]) was applied to account for experimental data obtained. In addition, the rate constants obtained were also explained with those predicted from an empirical expression [3].

### Mathematical models for the formaldehyde-sulphite reaction

The mechanism of the formaldehyde-sulphite clock reaction with the formation of hydroxymethane sulphonate ($HMS^-$) and oxymethane sulphonate ($MS^{2-}$) is as follows [9]:

(*a*) Dehydration of methane glycol

$$CH_2(OH)_2 = HCHO + H_2O \qquad K_1 = k_1/k_{-1} = 5 \times 10^{-4} \quad (1)$$

(*b*) Nucleophilic attack on formaldehyde by sulphite ion

$$HCHO + SO_3^{2-} = {}^-OCH_2SO_3^-(MS^{2-}) \qquad k_2 = 5{\cdot}4 \times 10^{-6} \quad (2)$$

(*c*) Protonation of $MS^{2-}$

$${}^-OCH_2SO_3^- + H^+ = HOCH_2SO_3^-(HMS^-) \qquad K_3 = 6{\cdot}3 \times 10^{-11} \quad (3)$$

(*d*) Dissociation of bisulphite ion

$$HSO_3^- = SO_3^{2-} + H^+ \qquad K_4 = 6{\cdot}3 \times 10^{-8} \quad (4)$$

Reaction (1) is the rate-determining step. In these expressions, $K_1$ denotes the equilibrium constant of the reaction (1), while $K_3$ and $K_4$ represent the acid dissociation constants of $HMS^-$ and $HSO_3^-$, respectively; $k_i$ denotes the rate constant of the ith reaction. The clock reaction is first order with respect to formaldehyde and zeroth order to bisulphite or sulphite. Step (4) keeps the hydroxyl ion concentration low as long as sufficient amount of bisulphite is mantained. However, at the end-point, almost all bisulphite ions are consumed, resulting in a sharp increase in pH.

Warneck [9] obtained a quadratic equation for the variation of the hydrogen ion concentration with the formaldehyde-sulphite clock reaction. However, the formula proposed is not valid for systems with pH > 8. With the consideration of the dissociation of $HMS^-$ as shown in the reverse of reaction (3), the following cubic equation was obtained.

$$(q_a [H^+]^2 + q_b [H^+] + q_c)(K_3 + [H^+]) = 0 \quad (5)$$

with

$$q_a = [Na^+] - [S_{IV}]_0 + K_4$$

$$q_b = -K_4 \{(2 - E_{oc}) [S_{IV}]_0 - [Na^+] + K_w/K_4\} \quad (6)$$

$$q_c = -K_w K_4$$

$$[S_{IV}]_0 = [HSO_3^-]_0 + [SO_3^{2-}]_0$$

The symbol $E_{oc}$ as given in expression (5) represents the extend of conversion of sulphite (including the acid form, $HSO_3^-$) in the clock reaction and is defined as

$$E_{oc} = ([S_{IV}]_0 - [S_{IV}])/[S_{IV}]_0 = [HMS^-]/[S_{IV}]_0 \quad (7)$$

Equation (5) is used in the present work to simulate experimental pH profiles of the formaldehyde-sulphite reaction.

### Experimental

### *Sample preparation*

The reagents including sodium metabisulphite (Riedel-de Haën (RDH) Aktiengesellschaft, Wunstorfer Straße, D-3016 Seelze 1, Germany), disodium ethylenediamine-

0142–0453/92 $3.00 © 1992 Taylor & Francis Ltd.

tetraacetate (EDTA) (BDH, AnalaR, Broom Road, Poole, Dorset, BH12 4NN, England), anhydrous sodium sulphite (Beijing Chemical Works, Huagong Lu, Dongjiao, Beijing, China), and 36% formaldehyde (China National Chemicals, Xijiao, Erligou, Beijing, China) were obtained commercially of CP grade except those specified and were used without further purification. Double deionized water was utilized for solution preparation. The formaldehyde solution was standardized through the conventional method [11]. For reaction mixtures, fixed concentrations of formaldehyde (a) $1{\cdot}41 \times 10^{-2}$ M and bisulphate (b) $1{\cdot}12 \times 10^{-2}$ M, and different concentrations of sulphite solutions (c) were used with the presence of $7{\cdot}79 \times 10^{-4}$ M EDTA to give the b/c ratios equal to 1·0, 8·0 and 14·7 [1]. Another set of mixtures with identical compositions were used without the addition of EDTA in order to study the effect of aerial oxidation on the sulphite ion. The experimental procedure as adopted in this work is similar to those given by Burnett [1]. In all measurements, the temperature was kept at 25 °C by using a thermostated water bath.

*Interfacing hardware and software*

A computerized pH monitoring system with an IBM PC/XT interfaced to an Orion SA 720 pH/ISE meter via the RS232C protocol [12] was employed for data acquisition for the formaldehyde-sulphite reaction. Details about the hardware of the system are given in Chau [10].

Programs for data acquisition and treatment were coded using ASYST [13]. ASYST provides real-time display facilities and multi-display of paired data in numerical analyses. Thus the variation of pH with time can be easily monitored.

A program CLOCKAQC.ASY was developed to acquire pH data for the formaldehyde-sulphite reaction with the interrupt mode for the RS232C protocol being adopted. Paired time and pH data was obtained every 1·37 s and was displayed simultaneously on screen.

*The response rate of pH electrode*

An Orion 9102 BN research grade combination electrode was used in the pH measurements. The response rate of the electrode was checked by using the dipping method [14]. Firstly, a reference solution was prepared by mixing together products of the clock reaction. The Orion pH probe was immersed in the solution for a period of time and the equilibrium pH was measured. Afterward, the electrode was transferred to the mixture solution with reactants but with the absence of formaldehyde. After 30s the electrode was returned to the reference solution. The corresponding change in pH was recored continously to within ± 0·01 unit of the equilibrium value using the computerized pH measuring system. The procedure was repeated several times in order to investigate the reproducibility of the electode response.

*Simulation of pH profiles*

A pH-time plot give the clock time $t_c$ at the sharp upturn. The rate constant $k_1$ as shown in reaction (1) correlated to the clock, time by [7]

$$k_1 t_c = \ln [a/(a - b)] \qquad (8)$$

Since these two quantities vary with temperature, the pH – $E_{oc}$ plot is preferred for data treatment. A program CONVER.ASY developed in this work was implemented to convert the time factor into the corresponding $E_{oc}$ quantity through expression:

$$E_{oc} = a[1 - \exp(-k_1 t)]/(b + c) \qquad (9)$$

A program called CUBICWS.ASY as coded was then used to compute concentrations of hydrogen ion at given values of $E_{oc}$ through equation (5). Theorectical pH profiles thus obtained were compared with observed data. Listings of the programs CLOCKAQC.ASY, CONVER.ASY, and CUBICWS.ASY can be obtained from the authors upon request.

### Results and discussions

*Electrode response and error estimation*

The response rate of the Orion pH electode was investigated by using the formaldehyde-sulphite solution with a concentration ratio of b/c = 1·0. This solution composition should proceed the largest and the most rapid pH change of the various reactions being studied. Figure 1 shows four experimental electrode response

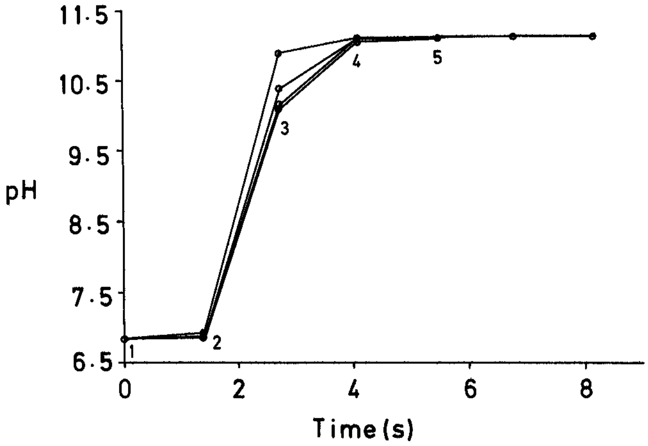

*Figure 1. Response curves of the Orion pH electrode. A time interval of 1·37 s was adopted for successive measurements.*

curves obtained from the dipping method. In these plots, the time interval between two consecutive points is 1·37 s, the minimum time required for the pH system to acquire a reading. After being immersed into the reference and the mixture solutions, the electrode was dipped into the reference solution again where measurements started at point 1. The interval between points 1 and 2 for the dipping step should not be included in continuous measurement. After point 2, the pH readings rose sharply at a rate of 2·54 ± 0·33 pH/s between points 2 and 3. The rate then dropped to 0·53 ± 0·37 pH/s for the next time interval. Finally, the pH values attained the equilibrium value of 11·13 ± 0·01 unit at point 5.

Rechnitz and Hameka [15] proposed an expression for cation-sensitive glass-electrode response in continuous measurements. However, it was found that the response curve of the Orion pH probe did not follow predictions from their expression probably owing to the nature of electrodes involved.

Table 1 lists the response time of the Orion pH sensor – three successive intervals of 4·11 s (= 3 × 1·37 s) from points 2 to 5 were required for the glass electrode to attain an equilibrium value. In continous pH recording, a total time lag of 4·11 s is required for the computerized measuring system in obtaining one datum accurately. Hence, the maximum error introduced in the pH-time profile of the clock reaction under study would be the corresponding change in pH at a given time and 4·11 s later. In this way, the relative errors for pH measurements can be estimated.

*pH-time profiles for the clock times*

Figures 2(*a*), (*b*) and (*c*) give the experimental pH-time profiles for the formaldehyde-sulphite reaction with the addition of EDTA at three different bisulphite/sulphite concentration ratios (b/c) of 1·0, 8·0 and 14·7 respectively. With the presence of appreciable amount of bisulphite ions, pH rises slowly with time as reaction proceeds. However, when the bisulphite concentration decreases to certain extent, the pH of the reaction mixture increases abruptly, as shown in Figure 2, and then becomes constant at the completion of the reactions. It is interesting to note that the initial pH value of a reaction mixture varies with the relative amount of bisulphite and sulphite present. The smaller the b/c ratio of a system (or the more the amount of sulphite added), the higher will be the initial pH value.

The errors introduced in the pH-time plots for the formaldehyde-sulphite reaction can be estimated using the method suggested previously for continuous measurements. Plots (d) to (f) of the calculated relative pH error (in %) against time corresponding to the pH profiles (a) to (c) respectively are shown in figure 2. For systems with b/c = 1·0, 8·0 and 14·7 respectively, the first 90%, 93% and 96% of the processing time before completion of the reaction have errors of less than 3% and the errors increase to a maximum of 18%, 16% for the remaining time.

The clock times of the formaldehyde-sulphite reaction were determined with the consideration of the final pH values to be within ±0·01 unit of the equilibrium values. The time lag factor for the electrode response had been considered in evaluating these quantities. Then values for $k_1$ were determined through equation (8). Table 2 lists the clock times, the rate constants $k_1$, and the final pH values thus determined for three different concentration compositions of the formaldehyde-sulphite reaction at 25 °C. The maximum error estimated for the clock times determined is ±3%. As mentioned previously, concentrations of formaldehyde and bisulphite solutions were kept constant in the study, only the sulphite concentration varied for different reaction mixtues. The one with b/c = 1·0 has the highest amount of hydroxide ion at the start of the reaction (figure 2). Since both sulphite and hydroxide ions catalyse the dehydration of methylene

*Table 1. Response time of the Orion pH electrode.*

| Interval between different points | Rate of pH change (pH/s) | Response time(s) measured from point 2 | Deviation from equilibrium pH value: pH | % error |
|---|---|---|---|---|
| 2–3 | 2·45 ± 0·33 | 1·37 | ±0·78 | 7·0 |
| 3–4 | 0·53 ± 0·02 | 2·74 | ±0·04 | 0.4 |
| 4–5 | 0·02 ± 0·02 | 4·11 | ±0·01 | 0·1 |

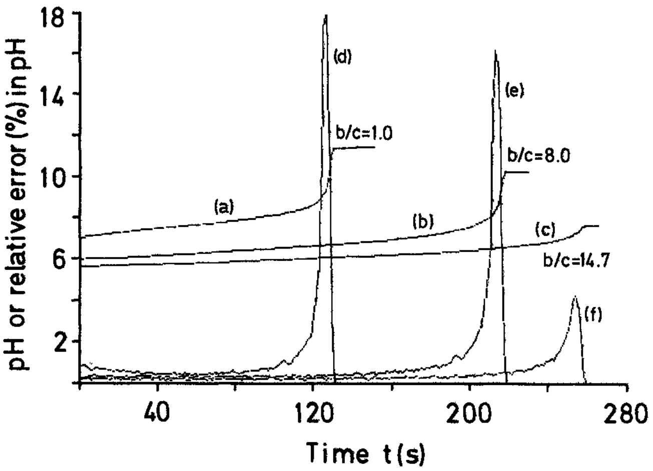

*Figure 2. The experimental pH profiles (a), (b) and (c), as well as the relative pH error curves (d), (e) and (f), in the presence of EDTA for the formaldehyde-sulphite reaction with different concentration ratios b/c of 1.0, 8.0 and 14.7 at 25 °C respectively.*

*Table 2. Clock times $t_c$ (s)[1], final pH values[1] and rate constants $k_1$ (x $10^{-3}s^{-1}$) for the formaldehyde-sulphite clock reaction at 25 °C*

| b/c ratio of the reaction mixture | $t_c$ corrected for time lag | Final pH value | $k_1$: Experimental[1,2] | $k_1$: Calculated[3] |
|---|---|---|---|---|
| 1·0 | 128·75 | 11·40 | 12·28 | 10·47 |
| 8.0 | 219·59 | 10·26 | 7·20 | 8·00 |
| 14·7 | 259·74 | 7·61 | 6·09 | 5·31 |

1. The relative errors of these quantities are ±3%.
2. The rate constants were derived from equation (5) – see text.
3. The rate constants were deduced through equation (10) – see text.

glycol (reaction (1)) [3], the rate constant of the clock reaction decreases for the series of mixtures with ratios b/c = 1·0, 8·0 and 14·7 as listed in table 2.

*Simulation of experimental pH profiles*

Experimental and theoretical pH − $E_{oc}$ plots for the formaldehyde-sulphite reaction with the b/c ratio having values of 1·0, 8·0 and 14·7 are given in figure 3. In general, the simulated curves generated from the cubic equation

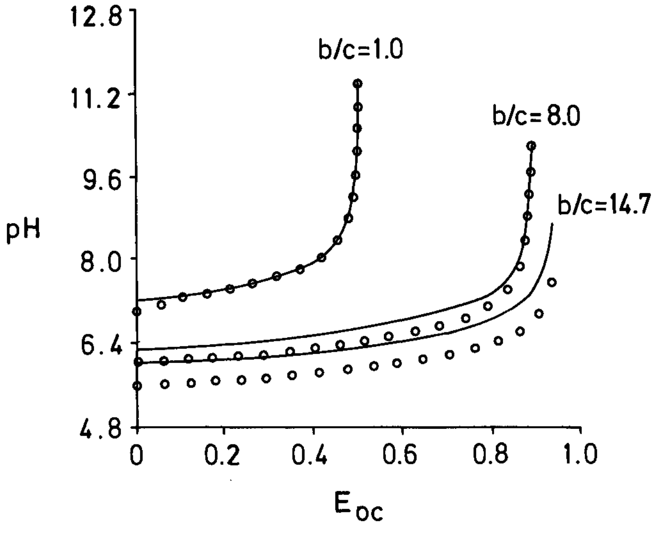

*Figure 3. The simulated (——) and experimental pH—$E_{oc}$ plots derived from equation (5) ( O O O ) with the presence of EDTA for the formaldehyde-sulphite reaction with the b/c ratios being equal to (a) 1·0, (b) 8·0, and (c) 14·7.*

(5) fit the experimental data well. For the reaction mixture with b/c = 1·0, the theoretical plot obtained from equation (5) agrees almost perfectly with the experimental one, with only a small deviation appears at the beginning of the reaction. This arises from the residual oxygen dissolved in the deionized water. A small amount of sulphite ions are oxidized by the oxygen to give sulphate ions whose conjugate acids are more acidic than those of the bisulphite. Therefore, the observed initial pH of the mixture is lower than the theoretical value in which no such factor being considered. The effect of aerial oxidation of sulphite ions becomes more prominent with the amount of sulphite ion being decreased for systems with a higher b/c ratios. This explains why discrepancies between the calculated and the observed pH values at the beginning, as well as in the middle of the clock reactions, increase with the b/c ratios (see figure 3).

In all cases with different b/c ratios, equation (5) gives no physical acceptable roots (or pH) beyond the clock times of the reactions. When $t = t_c$, a limiting value of $E_{oc}$ is obtained from equation (5) for the theoretical pH—$E_{oc}$ plot. This indicates that the reaction is completed at this time interval. The simulated values of $E_{oc}$ at $t_c$ matches the observed data – see figure 3. From the experimental point of view, no more bisulfite ions are available in reaction (4) around the end-point to maintain pH of the system and hence lead to a sharp rise in pH. In high concentration of $OH^-$ ions, hydroxymethane sulphonate ($HMS^-$) dissociates to give oxymethane sulphonate ($MS^{2-}$) in reaction (3) and, in turn, the chemical generates back the formaldehyde and sulphite ions in the reverse of reaction (2) [16]. Therefore, the formaldehyde-sulphite reaction stops shortly after the clock time and all the species involved in reactions (1) to (4) have their equilibrium values and $E_{oc}$ has its limiting value at this stage of experimentation.

Figure 4 gives the experimental pH profiles of the clock reaction with no EDTA being added. These profiles differ

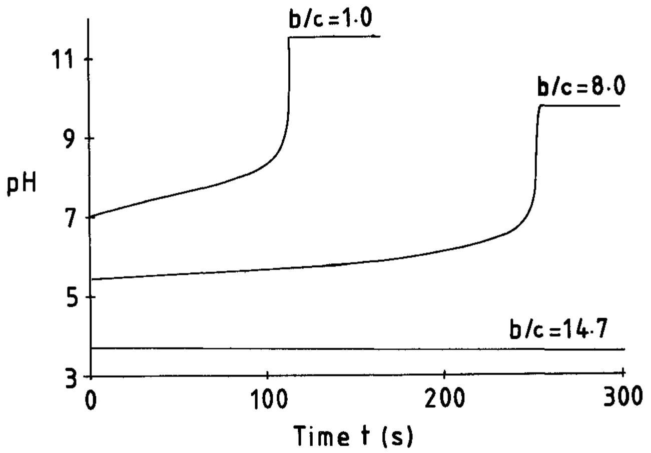

*Figure 4. The experimental pH profiles in the absence of EDTA for the formaldehyde-sulphite reaction with three different b/c ratios of 1·0, 8·0 and 14·7 at 25 °C.*

markedly from those with the presence of EDTA and give pH values lower than those as shown in figure 2 owing to aerial oxidation of sulphite as mention previously. In the case with b/c = 14·7, this effect becomes so serious that no clock time could be derived for the experimental data.

*Rate constant*

pH of the clock reaction keeps changing all the time during the clock reaction. The experimental $k_l$ values as listed in table 2 are average quantities within the whole process. According to Bell and Evans [2], the instantaneous rate constant $k_l$ (in $s^{-1}$) at 25 °C for the formaldehyde-sulphite reaction can be computed through the following expression:

$$k_l = 5{\cdot}1 \times 10^{-3} + 2{\cdot}7[H^+] + 1580[OH^-] + 0{\cdot}22[SO_3^{2-}] \quad (10)$$

By assuming that the concentrations of sulphite ions are constant throughout the reaction and using the experimental pH values obtained at different time intervals, instantaneous values of the parameter $k_l$ can be deduced through equation (10). Figure 5 shows the $k_l$-time plots

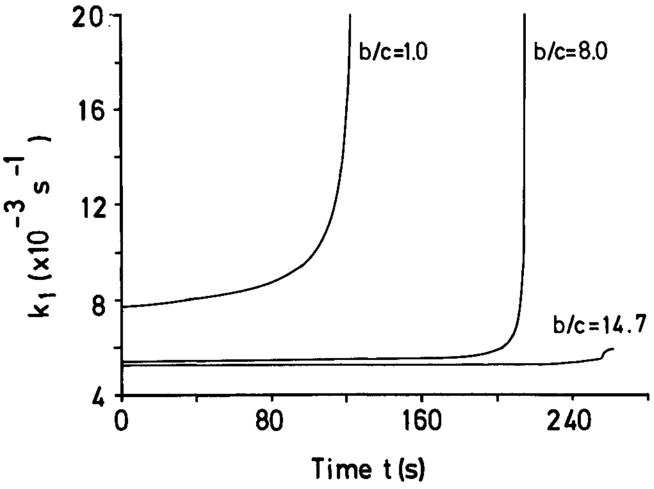

*Figure 5. The instantaneous rate constant $k_l$-time plots derived from equation (10) for the formaldehyde-sulphite reaction with three different concentration ratios b/c of 1·0, 8·0 and 14·7 at 25 °C.*

obtained in the manner. It can be seen that the initial rate for the system with b/c = 1·0 is high with respect to the other two systems with higher concentration ratio because reaction (1) is catalysed by the presence of sulphite ion. As the reaction proceeds, the concentration of $OH^-$ increases and the curve rises rapidly around the $t = t_c$ region (figure 5) resulting in very large $k_1$ values. Similar observation applies to the b/c = 8·0 system.

The average values of $k_1$ can be deduced by integrating the areas of the instantaneous $k_1$-time plots (figure 5) against time with interval between $t = 0$ and $t = t_c$ and then dividing the result by the clock time. In the present work, numerical integrations were carried out by the Simpson one-third method available in the ASYST software package [13] and $k_1$ thus derived via equation (10) are given in the final column of table 2 as the calculated values. Values of $k_1$ are to the experimental values, with small discrepancies due to the accuracy of the catalytic constants and the conditions under which the experiments were carried out. More accurate results can be obtained by using finer chemicals and an inert atmosphere.

### Conclusion

A computerized pH measuring system, including an IBM PC/XT and an Orion SA 720 pH/ISE meter, was used to monitor pH changes in the formaldehyde-sulphite clock reaction. Data acquisition and treatment were achieved through the use of ASYST.

A mathematical model based on the mechanism suggested by Burnett [1] was devised to explain experimental pH profiles at three different concentration compositions with success. The rate constants, $k_1$ and clock times for the reactions were correctly predicted. In addition, the $k_1$ values for different solution compositions were compared with those evaluated from the expression for instantaneous rate constant. From the present work, it has been shown that aerial oxidation of sulphite had marked effect on the reaction under study.

### Acknowledgement

This work was supported by grants from the Research Committee of the Hong Kong Polytechnic (Grant Nos. 341/023 and 341/510).

### References

1. Burnett, M. G., *Journal of Chemical Education,* **59** (1982), 160.
2. Bell, R. P. and Evans, P. G., *Proceedings of the Royal Society, Series* A, **291** (1966), 297.
3. Wagner, C., *Bev.,* **62** (1929), 2873.
4. Walker, J. F., *Formaldehyde* (Reinhold, New York, 1944), 29.
5. Sorensen, P. E. and Andersen, V. S., *Acta Chimica Scandinavia,* **24** (1970), 1301.
6. Cassen, T., *Journal of Chemical Education,* **53** (1976), 197.
7. Cooke, D. O., *Inorganic Reaction Mechanisms* (The Chemical Society Society, London, 1979), 71–75.
8. Boyce, S. D. and Hoffmann, M. R., *Journal of Physical Chemistry,* **88** (1984), 4740.
9. Warneck, P., *Journal of Chemical Education,* **66** (1989), 334.
10. Chau, F. T., *Computers in Chemistry,* **14** (1990), 69.
11. Welcher, F. J., *Standard Methods of Chemical Analysis,* Vol. IIB, 6th edn (USA, 1963).
12. Orion Research Inc., *SA720 Training Guide* (Orion, 529 Main Street, Boston, Massachusetts, 02129, USA, 1985).
13. Macmillan Software Co., *ASYST 2.0 Module 2 Manual* (Macmillan Software Co., 866 Third Avenue, New York, New York 10022, USA, 1987).
14. Rechnitz, G. A., *Talanta,* **11** (1964), 1467.
15. Rechnitz, G. A. and Hameka, H. F., *Z. Anal. Chem.,* **214** (1965), 252.
16. Morrison, R. T. and Boyd, R. N., *Organic Chemistry,* 3rd edn (India, 1973), 638–639.

